# A Sermon for the Kitchen

**Published:** 1890-03-08

**Authors:** 


### The Doctor's Pulpit.

### A SERMON FOR THE KITCHEN.

Domestic service of the feminine kind may be considered from the standpoint of the mistress, the maid, and the medical man. It is from the last of these three points of view that we shall here say a few words about it. The doctor who has had experience of a large family practice has necessarily come into wide contact with the servant class. A very important, a very wholesome, and a very pleasant class the present writer has on the whole found it to be. Both mistress and maid make some initial mistakes in their dealings one with another. Our purpose now is to point out to the maid some of her mistakes. Not long ago a young girl of seventeen said to her sister of sixteen something like this: "When you apply for a situation, take care that you ask exactly what your duties are to be, and when you go to the place see that you don't do anything except what you were engaged to do." Now it is not nice to call a girl of seventeen a "fool," but let us say that this young woman who thus advised her younger sister was at least a "goose," or more properly still a raw "gosling."

Does any young girl when she first makes up her mind for domestic service ever ask herself what she proposes to do, and be, as a domestic servant? When a boy of fourteen or fifteen enters upon his apprenticeship, it is probable he has some such thoughts as these in his mind: that he will do the best he can to learn his business quickly and to learn it well; that he will know how to do everything, both great and small, which belongs in any way to his trade. For if he has the very smallest grain of practical sense, he considers that the more he knows about his business the more likely he is to succeed in it; not only now when he is an apprentice, but in that happy future time when he shall have a business of his own. That is the young boy's view. But has not the girl as much sense as the boy? Has she not as much imagination? And does she not look forward to a happy future time when she, too, shall be mistress of her own house?

The girl, probably, has too decided a trick of looking a long way ahead, and so of thinking too little of the immediate present. The chief of all the faults we have to find with her is this, that she seems to have no idea whatever that domestic service for the first few years is her apprenticeship, exactly as the learning of a business is the apprenticeship of the boy. Is it possible by any means to get it into a girl's head that when she first goes out to service she is going to be an apprentice and to learn a business, a business by which she must live all the rest of her life, either in somebody else's house or in her own? But if she is going to learn a business, what a goose she must be if she makes up her mind to learn only a small part of that business, and to do as little as possible even of that small part. Ought not something like this to be the order of a domestic servant's training? She should first have a year or two in the kitchen at scrubbing and scouring; and when she has learnt to scrub and scour so thoroughly that not a single speck of dirt dare show itself within reach of her arm or her longest-handled broom, she may be considered to have mastered that part of her work pretty well. Then she should go upstairs and learn when to speak and when to be silent; to be as clean and look as neat as the newest pin that Birmingham ever produces; to keep the rooms in such spick and span order as no professional upholsterer could surpass or even equal; and to answer the door, where no manservant is kept, with a promptitude which lightning itself could not exceed. After two or three years diligently spent in this way, her upstairs education may be considered reasonably complete.

The most important part of domestic management is undoubtedly the cooking; and no servant, or mistress either, who has not an intelligent knowledge of cookery can be held to know anything at all adequate about the real business of housekeeping. The servant who has first scrubbed and scoured for two years in the kitchen, and then exercised her artistic talents for two more years upstairs, may properly be promoted to the highly honourable and deeply responsible post of assistant cook. Some rash and self-confident creatures will dare to call themselves "cooks" after two or three years in the scrubbing department. They might with as much propriety call themselves archbishops or physicians to the Queen. If it be true that a boy needs an apprenticeship of seven years to learn to be a grocer, then a girl should have an apprenticeship of fourteen years to learn to be a cook. That this is no exaggerated view of the situation is proved by the fact that whilst the best of grocers' assistants obtains no more than two pounds a-week as wages, the sublimest of cooks can command four or five times that amount.

Many unphilosophical persons, and none more than servants themselves, will think this subject quite unworthy of the grave consideration here given to it. But a doctor learns to take a different view of the importance of various occupations from that taken by most other men and women. The writer, for example, looks upon domestic service as one of the most important of all the callings in which women can be engaged. In his judgment it is much to be preferred to the work of shop assistants, barmaids, milliners, dressmakers, and all that class of occupations. A doctor of course attaches supreme importance to health; and domestic service in good families is infinitely more healthy than the miserable calling of the shop girl. Not only is the work less wearisome, but it tends much more to the exercise of the muscles and of the brain. The food, too, that domestic servants are supplied with is more comfortable and abundant; and what is quite as important, there is far more time for eating and digesting it. Domestic servants also are much more kindly and considerately treated by their employers than shop assistants are by their too often pompous, underbred, and tyrannical superiors. From a health point of view, domestic service is the best of all the occupations open to the women of the lower middle class.

Health is much, but it is not everything. Girls have to consider, or their parents for them, what the effect of a particular calling may be on the mind and the heart as well as on the body. Few, we think, who have studied the question will compare the calling of the shop assistant with the domestic servant's in this regard. The former finds it so difficult as almost to be impossible to preserve the wholesome and honest character with which she entered upon her calling. Lies have to be told sometimes instead of truth; tricks take the place of simplicity; a mean and sneaking deference to inferior persons kills and destroys wholesome self-respect. The domestic servant, on the other hand, has no temptation to tell lies, to practise tricks, or to fawn and cringe like a slave. The more truthful she is, the more honest and thorough in her work, the more self-respecting and modest in her behaviour, the more is she valued and the better is she paid. Foolish people may consider these things mere trifles, but they are trifles that often make the difference between virtue and ruin, between heaven and hell.

The subject is by no means exhausted. Space forbids to enter upon the consideration of other aspects of it at the present time. It, however, may be permitted to repeat that domestic service for women is a healthy, virtuous, and honourable calling; a calling eminently calculated to secure their immediate happiness and their future well-being, and the happiness and well-being also of their husbands and children, when in due time they marry and have homes of their own.

